# Symptomatological Features of Patients with and without Ecstasy Use during Their First Psychotic Episode

**DOI:** 10.3390/ijerph9072283

**Published:** 2012-06-27

**Authors:** Fabio Rugani, Silvia Bacciardi, Luca Rovai, Matteo Pacini, Angelo Giovanni Icro Maremmani, Joseph Deltito, Liliana Dell’Osso, Icro Maremmani

**Affiliations:** 1 Vincent P. Dole Dual Diagnosis Unit, Santa Chiara University Hospital, Department of Neurosciences, University of Pisa, Pisa 56100, Italy; Email: fabiorugani@libero.it (F.R.); silvia.bacciardi@libero.it (S.B.); lucarovai@yahoo.com (L.R.); 2 G. De Lisio Institute of Behavioral Sciences, Pisa 56100, Italy; Email: paciland@virgilio.it; 3 Association for the Application of Neuroscientific Knowledge to Social Aims (AU-CNS), Pietrasanta, Lucca 55045, Italy; Email: Deltito@aol.com; 4 Department of Psychiatry and Behavioral Science, New York Medical College, Valhalla, New York, NY 10019, USA

**Keywords:** Ecstasy use, psychotic acute episode, aggressive behavior

## Abstract

*Background*: Ecstasy use is generally chosen by adolescents and young adults for its entactogenic properties (the production of feelings of empathy, love, and emotional closeness to others.) Despite this desired and frequently realized outcome, Ecstasy use has often resulted in the genesis of psychotic symptoms and aggressive behaviors, particularly after chronic and/or intensive use. *Methods*: To explore the negative consequences of Ecstasy use and to examine the aggressive nature oftentimes seen in many Ecstasy users we employed a case-control study model. We compared, by means of validated psychometric tests, the psychopathological symptoms (BPRS), the aggressiveness (OAS) and the social adjustment (DSM-GAF) of psychotic patients with (n = 23) and without (n = 46) recent user of Ecstasy, during their first psychotic episode and hospitalization. All 23 Ecstasy users were Ecstasy users only. *Results*: Almost all of the psychotic symptoms were of similar severity in both groups. Blunted affect was milder in users than in non-users, whereas hostility and aggressive behavior was significantly more severe in users than in non-users. *Conclusions*: psychosis with a high level of aggressiveness and violence constitutes an important ‘side-effect’ that surely runs counter to the expected entactogenic action of Ecstasy. At a patient psycho-educational level, this study suggests that the use of Ecstasy may be counterproductive with respect to user expectations.

## 1. Introduction

Ecstasy first became available on the street in the late 70s, and in the 80s was called ‘queen of the designer drugs’ because of its widespread consumption and popularity. Social events known as ‘raves’ (all-night dance parties enhanced by upbeat electronic music and special lighting effects) were the main venue for the introduction of Ecstasy to many young people. Ecstasy’s popularity was a result of its ability to enhance pleasure, social intimacy, and emotional openness. Partygoers are motivated to use it primarily for its supposed energy-stimulating and euphoric effects [1]. Presently, especially youngsters use internet as a tool to increase their knowledge about Ecstasy effects and, as well, a mean to obtain the substance [2,3].

Unfortunately, the most common effects related to Ecstasy use include aggressiveness and violent behaviors, which has been explored in several inquiries [4]. Other authors have investigated whether the acute use of Ecstasy was associated with aggression that developed after its consumption [5,6]. Young people with a higher prevalence of lifetime Ecstasy use exhibited higher levels of aggressive and violent behavior [7]. Lastly, a higher level of violence seems to be present not only in active Ecstasy users, but in abstinent users as well [8]. Regrettably, these studies often fail to assess whether patients only used Ecstasy as their sole drug of abuse, or if they use other substances, a factor usually associated with severe outcome [9].

Psychotic symptoms attributable to the use of Ecstasy have been widely documented [10,11,12,13,14,15,16] with persecutory delusions as the most common presentation [17]. In one notable case acute paranoid psychosis was elicited after Ecstasy consumption, and was complicated by violence and legal consequences [18]. Similarly, one case has been reported of recurrent paranoid psychotic episodes in a patient with a history of Ecstasy abuse, characterized by an intense awareness of the personal need to make threats and carry out physical aggression, followed by abnormal corporal perceptions [19]. 

The relationship between use of Ecstasy and onset of psychosis has not been clearly established. Ecstasy could directly induce psychotic symptoms or act as a trigger on susceptible individuals. Patients who are already psychotic are probably more inclined to experiment with the substance [20]. Moreover, Ecstasy is frequently used by youngsters in whom psychopathological symptoms are going to occur regardless of the use of Ecstasy. The presence of a personal or family history of psychiatric disorders is important in determining the onset of the psychosis in Ecstasy users [17,21]. Other authors have reported cases of psychosis that have occurred in individuals who had no history of psychiatric disorders or positive precedents in their family [22,23]. Psychological complications following the use of Ecstasy appear to be rare, but when they do occur they are generally quite severe [24]. In some cases psychopathology persists even when the substance is no longer being taken [22,25,26]. Persistent psychosis is generally present in heavy, chronic abusers of Ecstasy, but some authors have documented cases in which psychotic symptoms have occurred after just one recreational dose of Ecstasy [24,27]. 

This study aims to confirm the association between Ecstasy, psychosis and aggressive behavior. To accomplish this we compared patients with acute psychosis related to the use of Ecstasy (in the absence of the use of other drugs and with a negative previous personal and familial psychiatric history) and acute psychosis patients with no record of resorting to substances of abuse.

## 2. Methods

A retrospective, naturalistic, epidemiological observational “case-control” study was designed. The research was implemented using a standard dataset recorded at the University Psychiatric Hospital in Pisa, Italy, including anonymous individual information originally collected for clinical care. The study included all psychotic patients who needed hospitalization after Ecstasy use only, during the period 2000–2011. All patients received a diagnosis of acute psychosis and gave their informed consent for the anonymous use of their personal data records for research purposes.

We selected “case” group patients according to the following criteria:

- Presence of an acute psychotic episode (1st episode)
- Absence of an history of psychopathological symptoms before starting using Ecstasy
- Positive urinary screening displayed concentration values which indicated use of Ecstasy in the previous week
- Negative urinary screening for opioid, cannabinoids, other stimulants, benzodiazepines and hallucinogens.
- Self reported negative history of substance (ab)use (other than Ecstasy)
- No problematic use of alcohol (self reported and confirmed by the principal household member).

The “case” group consisted of 23 male 20 ± 3 year old patients.

We selected “control” group patients according to the following criteria:

- Presence of an acute psychotic episode (1st episode)
- Demographic characteristics similar to those within “case group”
- Negative urinary screening for Ecstasy, opioid, cannabinoids, stimulants, benzodiazepines, hallucinogens
- Not reporting a past or current history of taking Ecstasy or any other substance of abuse
- No problematic use of alcohol (self reported and confirmed by principal household member).

The “control” group consisted of 46 male 21 ± 7 year old patients 

Discontinuation of tobacco use was not compulsory during hospitalization.

All patients were manifesting their first psychotic episode. These episodes had to be severe enough to require hospitalization. Most of the patients were single (N = 59; 85.5%) and unemployed (N = 37; 53.6%), and had experienced less than eight years of formal education (N = 37; 53.6%). Mean age was 21 ± 6 year old.

### 2.1. Instruments

#### 2.1.1. Brief Psychiatric Rating Scale (BPRS)

The BPRS, developed by Overall and Gorham [28], consists of 18 symptom constructs, each to be rated on a seven-point scale of severity ranging from “not present” to “extremely severe”. If a specific symptom is not rated, the figure 0 stands for “not assessed”. The BPRS provides a rapid and efficient evaluation of treatment response both in clinical drug trials and routine clinical settings. Its focus is primarily on inpatient psychopathology.

#### 2.1.2. Overt Aggression Scale (OAS)

The OAS, by Yudofsky and colleagues [29], is a 15-item observer evaluation rating scale comprising four factors: “verbal aggression” (four items), “clastic aggression” (four items), “self-aggression” (four items), “violence toward others” (three items).

#### 2.1.3. Global Assessment of Functioning (GAF)

Social adjustment was evaluated by means of the GAF [30]. The GAF scale reports the clinician’s judgment on an individual’s overall level of functioning. The maximum level (a score of 100) indicates efficient functioning over a wide range of activities; the minimum level (a score of one) indicates a persistent danger that the individual will hurt him/herself or others. Ten levels of functioning are available. Intermediate scores are used when appropriate.

#### 2.1.4. Psychiatric Diagnostic Evaluation

Diagnosis of “acute psychotic episode” was made on the basis of the DSM-IV Decision Trees for Differential Diagnosis [30]. Each decision tree starts with a set of clinical features. When one of these features is a prominent item of the current clinical picture, the clinician will ask a series of questions to rule in or rule out a number of disorders. The questions are just approximations to the diagnostic criteria that are used, and are not meant to replace them. Three decision trees have been used: “Differential Diagnosis of Psychotic Disorders” (initial clinical features: delusions, hallucinations, disorganized speech, or grossly disorganized behavior); “Differential Diagnosis of Mood Disorders” (initial clinical features: depressed, elevated, expansive or irritable mood; two separate items record the presence of depression and/or any tendency towards the bipolar spectrum as testified by an elevated, expansive or irritable mood); “Differential Diagnosis of Anxiety Disorders” (initial clinical features: symptoms of anxiety, fear, avoidance, or increased arousal).

#### 2.1.5. Urinalysis

Urine samples were collected at the time of hospitalization. 

We utilized the routine analyses as used for all hospitalized patients. Enzyme-multiplied immunotechniques were used for opiates, methadone, benzodiazepines, hypnotics, cocaine, amphetamines (including Ecstasy), hallucinogens, cannabinoids and inhalants. Problematic alcohol use was defined according to a lifetime history of frequent intoxication and/or negative consequences of habitual use on patients’ social adjustment (study/work, family, social/leisure or legal issues).

### 2.2. Statistical Analyses

Univariate comparisons between the two groups were evaluated by Chi-square analysis for categorical variables and by Student’s T-test for dimensional ones. Multivariate comparisons were evaluated by Discriminate Analysis using BPRS items and OAS dimensions (separately) as independent variables. Discriminate analysis is useful to statistically distinguish between two or more groups of cases. The mathematical objective of discriminate analysis is to weight and linearly combine the discriminating variables in some fashion so that the groups are forced to be as statistically distinct as possible. More importantly, the weighting coefficients can be interpreted much as in multiple regression or factor analysis. In this respect, they serve to identify the variables, which contribute most to differentiation along the respective dimension (function). All statistical analyses were performed using the statistical routines of SPSS (Statistical Package for Social Sciences) Version 20.0.

## 3. Results

Table 1 shows the demographic characteristics of our sample. The two groups showed similar demographic data. Education (<8 years) and current hospitalization (in days) both showed a shorter duration in Ecstasy users than in non-users. 

**Table 1:** Demographic and clinical data.

	Psychosis after Ecstasy use N = 23	Psychosis without substance abuse N = 46		
	N (%)	N (%)	Chi (df)	*p*
Marital status (single)	21 (91.3)	38 (82.6)	0.93 (1)	0.333
Education (<8 years)	18 (78.3)	19 (41.3)	8.42 (1)	0.003
Occupation				
Student	3 (13.0)	6 (13.0)		
White collar	1 (4.3)	5 (10.9)		
Blue collar	4 (17.4)	13 (28.3)		
Unemployed	15 (65.3)	22 (47.8)	2.35 (3)	0.502
Income (poor)	22 (95.7)	38 (82.6)		
Living situation (alone)	21 (91.3)	35 (76.1)	2.32 (1)	0.125
	M ± sd	M ± sd	T	*p*
Age	20 ± 3	21 ± 7	−0.89	0.377
Duration of hospitalization (days)	21 ± 12	48 ± 50	−2.80 *	0.005
GAF at baseline	22.21 ± 2.4	21.45 ± 4.6	0.89	0.379
GAF at discharge	40.17 ± 5.8	38.54 ± 9.4	0.88	0.382

* Man-Whitney U, Wilcoxon Rank Sum W Test (z score)

Table 2 shows the differences in BPRS profiles. All of the psychotic symptoms, except blunted affect and hostility, were of similar severity in both groups. In Ecstasy users, blunted affect was less severe and hostility more severe than in non-users. Despite that the total score of the BPRS could not differentiate between the two groups, hostility and blunted affect were able to correctly re-classify 85.5% of the patients, showing a satisfactory discrimination power.

**Table 2:** Most discriminant symptomatological characteristics of psychotic inpatients at start of hospitalization.

	Psychosis after Ecstasy use N = 23	Psychosis without substance abuse N = 46			
	M ± sd	M ± sd	F	*p*	Discriminant Function *
Brief Psychiatric Rating Scale					
10. Hostility	4.39 ± 1.0	3.09 ± 1.3	16.94	0.000	−0.44
16. Blunted affect	2.04 ± 1.5	3.52 ± 1.4	14.92	0.000	0.41
2. Anxiety	3.26 ± 1.2	3.98 ± 1.6	3.44	0.068	0.19
11. Suspiciousness	3.96 ± 1.3	4.52 ± 1.2	3.02	0.086	0.18
18. Disorientation	1.00 ± 0.0	1.13 ± 0.5	1.32	0.254	0.12
3. Emotional withdrawal	3.09 ± 1.8	3.45 ± 1.6	0.73	0.395	0.09
17. Excitement	2.96 ± 2.0	2.54 ± 1.7	0.76	0.384	−0.09
4. Conceptual disorganization	3.57 ± 1.4	3.85 ± 1.4	0.57	0.452	0.08
6. Tension	3.35 ± 1.7	3.61 ± 1.4	0.42	0.519	0.07
12. Hallucinatory behavior	2.61 ± 1.8	2.39 ± 1.9	0.20	0.656	−0.04
14. Uncooperativeness	4.17 ± 1.7	4.00 ± 1.4	0.19	0.662	−0.04
5. Guilt feelings	2.48 ± 1.3	2.59 ± 1.6	0.07	0.789	0.02
7. Mannerism and posturing	2.39 ± 1.5	2.48 ± 1.5	0.04	0.828	0.02
8. Grandiosity	2.30 ± 1.7	2.35 ± 1.6	0.01	0.920	0.01
13. Motor retardation	2.43 ± 1.6	2.50 ± 1.5	0.02	0.872	0.01
1. Somatic concern	2.65 ± 1.5	2.67 ± 1.5	0.00	0.957	0.00
9. Depressive Mood	2.43 ± 1.6	2.46 ± 1.3	0.00	0.954	0.00
15. Unusual thought content	4.83 ± 1.3	4.83 ± 0.9	0.00	1.000	0.00
centroids	−1.58	0.79			
	M ± sd	M ± sd	T	*p*	
Total BPRS	53.91 ± 8.9	55.95 ± 7.48	−0.94	0.353	

* Statistics: Wilks’s lambda 0.43 chi-square = 48.29 df 18 *p* < 0.001. Reclassification power 85.5%.

Table 3 shows the differences in OAS profiles. After Ecstasy use psychotic patients were more violent, clastic violent and verbal aggressive than non-users. No differences were observed regarding self-aggression. Discriminate function suggested that hetero-aggression (violence or assault) was more important than indirect aggression (clastic violence) to differentiate Ecstasy users from non-users.

**Table 3:** Most discriminate aggressive behaviors of psychotic inpatients at start of hospitalization.

	Psychosis after Ecstasy use N = 23	Psychosis without substance abuse N = 46			
	M ± sd	M ± sd	F	*p*	Discriminant Function
Overt Aggression Scale (OAS)					
Violence	1.83 ± 1.0	0.85 ± 1.1	11.53	0.001	0.94
Clastic violence	2.17 ± 0.9	1.30 ± 1.1	9.35	0.003	0.85
Verbal aggression	3.52 ± 0.5	3.00 ± 0.9	5.62	0.021	0.66
Self-aggression	0.35 ± 0.4	0.20 ± 0.5	1.16	0.285	0.30
centroids	0.61	−0.30			
	M ± sd	M ± sd	F	T	
OAS-Total	7.86 ± 2.4	5.34 ± 2.9	3.78	0.000	

Statistics: Wilks’s lambda 0.83 chi-square = 11.44 df 4 *p* = 0.022. Reclassification power 66.7%.

## 4. Discussion

All of our inpatients who show psychotic symptoms related to Ecstasy use are characterized by a less blunted affect that is, however, accompanied by hostility and aggressive behavior (typified by violence—in particular, assault and clastic violence). They also present a lower educational level and a shorter duration of hospitalization.

The role of blunted affect may be linked to two possible confounding factors: the first is the role of anhedonia, usually described in alcohol/substance abusers in post-detoxification conditions [31]. The second is the importance of the possible effect exerted by the use of neuroleptics, which should be adequately controlled for the possibility to cause side effects, such as emotional blunting and other neurological soft signs [32]. Both this conditions are not present in our sample.

In our sample the low levels of education might have increased the risk of Ecstasy use. The shorter duration of hospitalization argues instead for a lower severity of psychosis compared with spontaneous psychosis. This seems true at least with regard to the efficacy of standard anti-psychotic treatments.

The correlation between aggressive-behavior and Ecstasy use is consistent with a large body of literature. It is well known that low quantities of serotonin and its metabolites play an important role in modulating impulsive violence and self aggression [33,34,35,36,37,38]. Moreover, the direct effects of Ecstasy and a hypo-serotoninergic state may subsequently cause dopaminergic hyperactivity, which is most likely responsible for the onset, sometimes not for the first time, of acute psychotic symptoms [25]. Consistently with the present study, previous reports shed light on the more aggressiveness and violent behaviors of psychotic Ecstasy users [18,19]. 

In summary, acute psychotic users showed “less blunted affect” and more “violence toward others” and “clastic aggression” than psychotic non-users; “autolesionism” was present in equal frequency in the two groups. This study emphasizes that an aggressive psychosis can follow the use of Ecstasy even without the use of other substances of abuse and with a negative psychiatric history, causing an effect opposite to what young people, generally express they want to obtain by using Ecstasy. Interestingly, the same symptomatological features (*i.e.*, presence of less blunted affect, more clastic aggression and violence towards others) have also been observed in psychotic cannabis users [39]. Cannabis users often say they use cannabis for its entactogenic effects.

Limitations of the present study are the following:

1. The sample of psychotic Ecstasy users consisted entirely of males, because it derives from a data set recorded at a male ward. As a result this psychopathological considerations cannot be applied to samples comprising both genders. Nevertheless, no gender related difference as been reported in Ecstasy users regarding aggressive behavior [6].
2. The retrospective design of the study did not allow us to consider some quantitative variables such as the amount of Ecstasy tablets ingested in the life time or on the last Ecstasy intake occasion.
3. SCID-I for the diagnosis of axis-I DSM-disorders may be more appropriate.
4. The absence of a history of substance abuse other than Ecstasy is self-reported; anyway no other substances were detected at patients’ hospitalization.

## 5. Conclusions

In our asymptomatic patients the use of Ecstasy is followed by psychosis and increasing impulsive and violent behaviors. In the Ecstasy users the less blunted affect can be due to the absence of “pure” psychosis rather than consequence of substance use, but the high level of aggressiveness and violence represents an important ‘side-effect’ of Ecstasy action. On clinical grounds, this study supports the good clinical practice, when patients are receiving counseling, to stress the concept that the use of Ecstasy may be counterproductive to the expectations of the subject. 
